# Risperidone-Loaded PLGA–Lipid Particles with Improved Release Kinetics: Manufacturing and Detailed Characterization by Electron Microscopy and Nano-CT

**DOI:** 10.3390/pharmaceutics11120665

**Published:** 2019-12-09

**Authors:** Christopher Janich, Andrea Friedmann, Juliana Martins de Souza e Silva, Cristine Santos de Oliveira, Ligia E. de Souza, Dan Rujescu, Christian Hildebrandt, Moritz Beck-Broichsitter, Christian E. H. Schmelzer, Karsten Mäder

**Affiliations:** 1 Institute of Pharmacy, Faculty of Biosciences, Martin Luther University Halle-Wittenberg, 06120 Halle (Saale), Germany; christopher.janich@pharmazie.uni-halle.de (C.J.); ligia.souza@pharmazie.uni-halle.de (L.E.d.S.); 2 Department of Biological and Macromolecular Materials, Fraunhofer Institute for Microstructure of Materials and Systems IMWS, 06120 Halle (Saale), Germany; andrea.friedmann@imws.fraunhofer.de (A.F.); christian.schmelzer@imws.fraunhofer.de (C.E.H.S.); 3 Institute of Physics, Martin Luther University Halle-Wittenberg, 06120 Halle (Saale), Germanycristine.santos-de-oliveira@physik.uni-halle.de (C.S.d.O.); 4 Department of Psychiatry, Psychotherapy and Psychosomatics, Martin Luther University Halle-Wittenberg, 06120 Halle (Saale), Germany; dan.rujescu@uk-halle.de; 5 MilliporeSigma a Business of Merck KGaA, 64293 Darmstadt, Germany; christian.hildebrandt@merckgroup.com (C.H.); moritz.beck-broichsitter@merckgroup.com (M.B.-B.)

**Keywords:** controlled release, PLGA, risperidone, microparticles, microcapsules, oleogels, electron microscopy, three-dimensional X-ray imaging, nano-CT, biodegradable polymers, hydroxy-stearic acid

## Abstract

For parenteral controlled drug release, the desired zero order release profile with no lag time is often difficult to achieve. To overcome the undesired lag time of the current commercial risperidone controlled release formulation, we developed PLGA–lipid microcapsules (MCs) and PLGA–lipid microgels (MGs). The lipid phase was composed of middle chain triglycerides (MCT) or isopropylmyristate (IPM). Hydroxystearic acid was used as an oleogelator. The three-dimensional inner structure of Risperidone-loaded MCs and MGs was assessed by using the invasive method of electron microscopy with focused ion beam cutting (FIB-SEM) and the noninvasive method of high-resolution nanoscale X-ray computed tomography (nano-CT). FIB-SEM and nano-CT measurements revealed the presence of highly dispersed spherical structures around two micrometres in size. Drug release kinetics did strongly depend on the used lipid phase and the presence or absence of hydroxystearic acid. We achieved a nearly zero order release without a lag time over 60 days with the MC-MCT formulation. In conclusion, the developed lipid-PLGA microparticles are attractive alternatives to pure PLGA-based particles. The advantages include improved release profiles, which can be easily tuned by the lipid composition.

## 1. Introduction

The use of parenteral controlled release drug delivery systems (CR-DDS) is a highly attractive and rational way to improve drug therapy. Potential benefits include decrease of administration frequency, increase of bioavailability and less side effects. Polylactic acid (PLA) and poly(lactic-*co*-glycolic acid (PLGA) products have existed for several decades and are, by far, the most widely used polymers for CR-DDS [1,2]. The major clinical use is the peptide-based treatment of GnrH-dependent diseases such as breast and prostate cancer. Other important and increasingly used applications include the local delivery of drugs to the eye [3], the brain [4], or the inner ear [5,6]. An important application of CR-DDS is also the controlled release of antipsychotic compounds to treat schizophrenia. Long-acting parenteral controlled release of antipsychotic drugs overcomes the poor compliance of patients with oral administration [7,8]. Risperidone is an important drug in the treatment of schizophrenia. Several oral and one parenteral controlled release formulations are available and a PLGA based CR-DDS formulation is marketed (Risperdal^®^ Consta^®^). According to the treatment schedule [9], patients initially have to take tablets, although the CR-DDS has already been injected. This situation is not a favorable scenario of pharmacotherapy, because schizophrenia patients do frequently not comply and double medication should be avoided. The release mechanisms of Risperdal^®^ Consta^®^ have been studied in detail by the group of Burgess and good in vitro and in vivo correlations have been observed by means of scaling factors [10,11]. Initially, a lag phase with almost no release is observed, followed by a faster release. The group clearly showed that the release onset is related to the water penetration and polymer degradation-induced drop of the polymer glass transition temperature *T*_g_ to 37 °C.

Several publications describe alternative PLGA formulations for the controlled release of risperidone. However, commonly, the presence of lag times, sigmoidal release curves, and rather short release times (<1 month) have been observed [10,11,12,13,14,15]. Souza et al. tested different formulations with single or repeated doses in vivo [16]. For single dose injections, peak maxima were observed after around one week. Unfortunately, no in vitro release data were published in the paper [16]. Therefore, it is clearly still desirable to develop biodegradable DDS with better risperidone release kinetics. The CR-DDS should provide a constant release over the 1–2 months without a lag time and the need for initial oral comedication. It should also be cost effective. Unfortunately, the selection possibility for biodegradable materials for parenteral controlled release is rather limited. In addition to PLA and PLGA, there are few other polymers and lipids. Liquid lipids are used clinically, but they provide only release control for a short period of few days. Solid lipids are also attractive as parenteral depot systems [17], but their use is often linked with problems of polymorphic transitions and limited enzymatic degradability due to the crystalline structure of the lipid. The use of polymer–lipid hybrid structures—compared to pure polymer or pure lipid-based DDS—offers additional opportunities. For example, increased drug loads may be achieved by the lipid component and the release rate might be controlled by the polymer shell. The incorporation of liquid oil into a microparticle might compromise the mechanical stability of the particle and could potentially lead to oily leakage and loss of release control. The incorporation of solid lipids will overcome this problem, but this advantage is at the cost of drug solubility in the crystalline lipid matrix and the problem of polymorphic transitions. It is desirable to combine the advantages of a liquid lipid phase with high mechanical stability. Therefore, we decided to investigate oleogels as possible alternatives. A suitable oleogelator for parenteral applications is 12-hydroxystearic acid (HS). Hydroxystearic acid induces gel formation in lipids due to its precipitation as a HS-fiber network [18,19]. Preclinical studies on injectable oleogels demonstrate the good biocompatibility and degradability of HS-oleogels in vivo [20,21].

We selected PLGA Expansorb^®^ 75-7E from Merck KGaA (Darmstadt, Germany), which has the following properties: lactide/glycolide (L/G) ratio of 75/25, a molecular weight (MW) 80–115 kDa, and the end groups are esterified. The physical chemical properties of Expansorb^®^75-7E were similar to the PLGA in the market product Risperdal Consta^®^ which contains a lactide/glycolide (L/G) ratio of 78/22, a molecular weight (MW) ~111 kDa and the end groups are esterified [22]. As the oil phase, we incorporated isopropyl myristate (IPM) or middle chain triglycerides (MCT) to form a microcapsule (MC) structure. Oily components with saturated fatty acids were used to avoid the danger of oxidation problems connected with unsaturated fatty acids.

**Figure 1:**
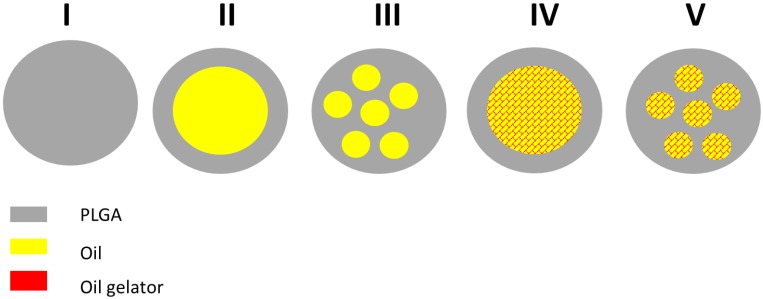
Possible structures of poly(lactic-*co*-glycolic acid (PLGA) and PLGA/lipid microparticles: I: PLGA microparticle; II: microcapsule with core–shell structure; III: microcapsule with multiple oily droplets; IV: microgel with core–shell structure; microgel with multiple gelled lipid domains.

The properties and the release kinetics of the micro-DDS will depend on the general structure and the physicochemical state of the drug and the excipients. Possible structures of (drug-free) micro-DDS are shown in Figure 1.

PLGA microparticles are expected to form a homogeneous polymer matrix (structure I). Microcapsules composed of PLGA and oil could show structures with a single oily core and a PLGA shell (structure II) or multiple oily droplets encapsulated in PLGA (structure III). The addition of the oil gelator will increase the viscosity, but again, the oily phase could exist as a single core (structure IV) or as multiple cores (structure V). The drug could be dissolved in the polymer and the oil and/or exist as an own solid (crystalline or amorphous) phase. A rational development of the DDS requires an appropriate characterization. For particle sizing of micro-DDS, static light scattering is the standard method. We decided to use X-ray diffraction for the detection of crystalline structures in our DDS. In addition, we wanted to explore the microstructure inside the DDS. For this purpose, we used two complementary imaging techniques, which were focus ion beam scanning electron microscopy (FIB-SEM) and Zernike phase-contrast X-ray nano-computed tomography (nano-CT). By means of focused ion-beam preparation, we could cut the particles in situ and we were able to visualize the morphology of the microstructure at the location of the cut. In addition, we performed nano-CT experiments. Due to the high penetration depth of X-rays, nano-CT provided high-resolution three-dimensional images of the DDS and we were able to visualize their internal structures at any virtual slice without the need for cutting or slicing them.

In summary, the main goals of this work were:

(i) development of risperidone loaded PLGA–lipid microcapsules (MCs) and PLGA–lipid microgels (MGs) with an optimized release profile (no lag time, constant release rate)
(ii) characterization of the three-dimensional inner structure of risperidone-loaded MCs and MGs by using focused ion beam (FIB) preparation and three-dimensional X-ray imaging (nano-CT).

## 2. Materials and Methods

### 2.1. Materials

Expansorb DLG 75-7E, batch number C100010962, (Merck, Darmstadt, Germany) PVA 4-88 (Merck, Darmstadt, Germany) Risperidone 95+% (Activate Scientific, Shanghai, China), Pioneer MCT (middle chain triglycerides) batch number: 1132229 (Hansen & Rosenthal, Hamburg, Germany); isopropylmyristate Ph-Eur. batch number: 1407015-01, (Euro OTC Pharma GmbH, Bönen, Germany); dichloromethane 99.9% (Carl-Roth, Karlsruhe, Germany); hydroxystearic acid was a gift from Alberdingk Boley GmbH, (Krefeld, Germany). Risperdal Consta^®^ 50 mg batch number: GCSK002, (Janssen-Cilag GmbH, Neuss, Germany); 2.5 mL GASTIGHT^®^ syringe (Hamilton Germany GmbH, Planegg, Martinsried, Germany), syringe pump (Pump 11 Elite, Harvard Apparatus, Holliston, MA, USA).

### 2.2. Methods

#### 2.2.1. Preparation of PLGA Microcapsules (MC) and PLGA Microgels (MG)

Risperidone-loaded microcapsules were prepared using an oil-in-water (o/w) emulsion solvent extraction/evaporation technique.

For this purpose, 100 mg PLGA 75-7E, 100 mg of the oil phase (MCT or IPM), and 50 mg of Risperidone were weighted into a 4 mL glass vial and dissolved into the final volume of 2 mL dichloromethane by vortexing. The organic solutions were drawn up into a 2.5 mL GASTIGHT^®^ syringe (Hamilton Germany GmbH, Planegg, Martinsried, Germany) and injected with a flow rate 5.2 µL/min by a syringe pump (Pump 11 Elite, Harvard Apparatus, Holliston, MA, USA) directly into 300 mL of aqueous PVA 4-88 (Merck, Darmstadt, Germany) solution 0.25% (*w/v*) stirred with a Rotating Paddle, USP at 240 rpm at room temperature. The aqueous phase contains double distilled and filtrated water with 0.25% polyvinyl alcohol PVA 4-88 (Merck, Darmstadt, Germany). When particle formation was completed, the suspensions were transferred into a 500 mL round bottom flask and the remaining DCM were removed under vacuum 40 mbar for 20 min. Thereafter, sedimented microcapsules were transferred by a glass pipette into a 15 mL tube and the supernatants were removed carefully. Then, the microcapsules were washed thrice with sterile filtrated double distilled water, filled up to the final volume of 1 mL and lyophilized.

#### 2.2.2. Lyophilization

For freeze-drying, the samples were frozen rapidly with liquid nitrogen at −196 °C and lyophilised on a Christ Alpha 2–4 freeze dryer (Martin Christ Gefriertrocknungsanlagen GmbH, Osterode am Harz, Germany) in combination with a Vacuubrand RC 6 vacuum pump (Vacuubrand GmbH, Wertheim, Germany) for 24 h. The chamber was evacuated to 0.05 mbar, corresponding to −48 °C on the sublimation curve of ice. The prepared microcapsules were stored at −20 °C until further use.

#### 2.2.3. Static Light Scattering Measurements

Routine measurements of the size distribution were carried out by laser diffractometry on a Mastersizer 2000 (Malvern Instruments, Malvern, United Kingdom) combined with a Hydro 2000 S wet dispersion unit. The samples were measured at a laser obscuration of 5%, corresponding to 11%–15% obscuration of the blue laser, in purified water. A series of five runs was evaluated by the Mastersizer 2000 software version 5.60, using the Mie theory, assuming spherical particles with a refractive index of 1.44, absorption of 0.001, and a refractive index of 1.33 for the dispersant as optical properties.

#### 2.2.4. Powder XRD

XRD analysis was performed on a Bruker D8-Advance diffractometer, equipped with a one-dimensional silicon strip detector (LynxEye) operating with Cu Kα radiation. Diffraction was measured from 2*θ* = 5° to 70° with a step size of 0.01° and a counting time of 1 s.

#### 2.2.5. High-Performance Liquid Chromatography (HPLC)

A HPLC Agilent 1100 Series (Agilent, Santa Clara, CA, USA) was used. Separations were carried out using a BDS-Hypersil^®^ C18 5 μm 250 × 4.6 column (Thermo Fisher Scientific, Waltham, MA, USA) and a mobile phase of 75% (*v/v*) methanol/25% (*v/v*) water, 1% (*v/v*) tetramethylammoniumhydroxid pentahydrate solution 25% *w/v*. Runs were carried out at 0.5 mL/min over 16 min and the absorption at 280 nm was recorded. The retention time of risperidone was 7.5 min. Injection volumes of 20 μL were used for determination of drug loading.

#### 2.2.6. Drug Loading and Encapsulation Efficiency (EE)

Risperidone-loaded microcapsules were weighed and diluted in methanol to get a final concentration of 0.5mg/mL. All the samples were sonicated for 10 min to accelerate the dissolving of MC. Thereafter all samples were diluted 0.05 mg/mL and filtered (Minisart SRP 4, 0.45 μm syringe filter, Sartorius Stedim Biotech GmbH, Göttingen, Deutschland). The concentration of risperidone was determined via HPLC. Drug loading and EE were calculated as following equations from literature: Drug loading = (weight of drug entrapped/weight of MC used) × 100%; EE = (experimental drug loading/theoretically drug) × 100% [23].

#### 2.2.7. In Vitro Drug Release Assay

The in vitro release of risperidone from MCs was carried out in a heated bath shaker (Memmert GmbH + Co. KG, Schwabach, Germany) at 37 °C and constant shaking at 50 rpm. A total of 6 mg of formulation I-IV and 3 mg of Risperdal Consta^®^ were suspended in 1 mL of 10 mmol phosphate buffer saline (PBS) at pH 7.4 and transferred into a Spectra Por Float-A_Lyzer ^®^ G2 1000kD. The float-A Lyzer was placed into a 100 mL measuring cylinder and filled up with PBS to the final volume of 60 mL so that the float-A Lyzer is submerged in PBS. To maintain sink conditions, the buffer solution was drawn off half (half change) when the risperidone concentration reaches 7%–8% of the solubility in PBS. Drug quantity was determined by UV-1800 spectrophotometer (Shimadzu, Kyoto, Japan). All measurements were performed in triplicate and the measurement points were represented as the mean ± SD.

#### 2.2.8. Scanning Electron Microscopy and Focused Ion Beam Preparation

High resolution investigations of the microparticle morphology were done by scanning electron microscopy (SEM) using a Quanta 3D FEG (field emission gun) instrument from FEI Company, (Hillsboro, OH, USA). The microparticles were carefully placed on SEM sample holders using carbon tape and subsequently coated with a thin layer of platinum using a magnetron sputtering system (HVD, Dresden, Germany) to achieve a conducting surface. The SEM images were obtained under high vacuum conditions using an acceleration voltage of 5 keV, a working distance of 6–10 mm and electron beam currents of 12 pA determined with an Everhart–Thornley detector.

The microscope is designed as a dual beam (electron and ion beam) which gives the possibility to perform FIB preparations in the same device. Here, the gallium ion beam and the electron beam operate independently of each other. The point of coincidence of the two beams is located at a working distance of 10 mm. The angle between both beams is 52°. To allow vertical cutting with the FIB, the sample was tilted by this angle. Thus observation of the cross-sections with the electron beam was also done at an angle of 52°. After screening the samples by conventional SEM, the particle of interest was selected for cross-sectional preparation (target preparation). As the first step in cross-section preparation at predefined areas of interest coarse material was ablated with gallium ions accelerated at 30 kV with ion currents of 15 nA. After this coarse milling step lower beam currents of Afterwards, 1–0.5 nA were used to polish the cross sections. The samples were not cooled during FIB milling. The patterning conditions used conform to the standard patterning conditions for silicon materials. The SEM observation can be done during ion milling or after subsequent milling steps.

#### 2.2.9. Nano-CT

X-ray imaging experiments were performed at the Institute of Physics, Martin Luther University Halle-Wittenberg, in a Carl Zeiss Xradia 810 Ultra (Cr source, 5.4 keV, for instrument details see [24]). Phase-contrast imaging mode was used with a phase-ring positioned near the back focal plane of the zone plate. In each experiment, a total of 901 projections with a field-of-view (FOV) of 65 μm were obtained over 180°, with an exposure time of 15 s per projection, a detector binning of 2, and voxel size of 128 nm. Image reconstruction was performed by filtered back-projection algorithm using the software integrated into the Xradia 810 Ultra. For each sample, two or three imaging experiments were performed, moving the sample vertically to enable the visualization of one entire microsphere in one single direction (from now on named Z-direction). For each sample’s datasets, the brightness and contrast were adjusted in ImageJ^®^ and the datasets were then stitched together using the Merge module of the Thermo Fisher Scientific Avizo^®^ software (version 9.2, Thermo Fisher Scientific, Hillsboro, OR, USA). Median denoising and nonlocal mean filter were applied to the datasets. The 3D renderings presented here were created using either the commercial software arivis Vision4D^®^ (version 2.12.6, arivis AG, Rostock, Germany) or Avizo^®^. For the dispersed internal spherical structures (ISS) evaluation, a volume located in the center of the 3D image was extracted consisting of 325 × 325 × Z voxels. Z varies among the samples, as it is almost equal to the number of pixels of the microsphere diameter, but is smaller than it to prevent voxels that do not consist of sample to be added to the calculation. The results obtained here represent the ratio of ISS compared to the total extracted volume of the sample (PLGA+ISS). For binarization of the datasets into PLGA and ISS, automatic thresholding with IsoData criterion was used, followed by island removal for the removal of small clusters of voxels (<15). A surface was generated from the segmented dataset in order to smooth sharp voxel edges and the specific surface area (SSA) values obtained represent the ratio of the constructed surface area (SA) to the volume (V) for each sample.

## 3. Results and Discussion

### 3.1. Preparation of MCs and MGs

For the preparation of MCs and MGs, the conventional oil-in-water (o/w) emulsion solvent evaporation method was used [2,25]. All components were dissolved in dichloromethane. The components of the four different formulations are listed in the Table 1. In general, the composition followed a 2:2:1 mass ratio of PLGA/Lipid/Risperidone. Therefore, the theoretical drug load was 20% [m/m]. High encapsulation efficiencies (>92%) were achieved, as seen in Table 1.

To get reproducible particle sizes, it was important to keep all size-influencing parameters constant, e.g., the o-phase volume and solvent, the concentration and type of polymer, the volume of the continuous phase and the type and concentration of stabilizer, the temperature, and the stirring speed [26]. After lyophilization the MCs did not agglomerate and were resuspendable in water.

### 3.2. Characterization

#### 3.2.1. Particle Size Measurements

**Figure 2:**
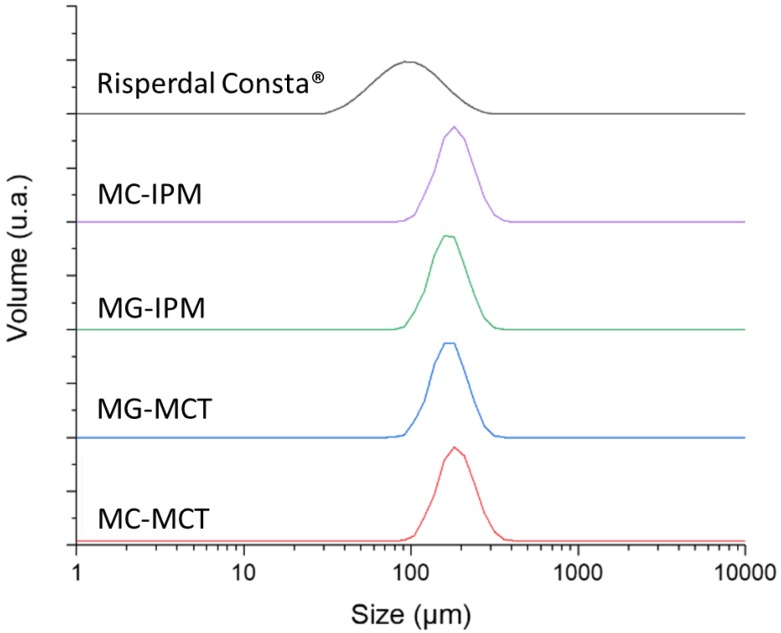
Volume-averaged particle sizes of microcapsules, microgels, and the reference product.

The particle size and morphology of MC are key factors which potentially affect EE, drug release rate, and the biodistribution [27,28]. The optimal size range for microparticles with controlled release characteristics is considered to be 10–200 µm. Particles in the lower micron range will show a faster drug release, and they are also more easily phagocytosed by immune cells [29]. Sizes larger than 200 µm are difficult to inject and will show more inhomogeneous degradation [30]. The particle size distribution is shown in Figure 2.

The particle size of the microcapsule formulations MC-IPM and MC-MCT are very similar (D_(0.5)_ = 172.8 µm and 169.5 µm). The particle sizes for the corresponding microgels MG-IPM and MG-MCT were smaller compared to the microcapsules, but again very similar between IPM and MCT (D_(0.5)_ = MG-IPM 157.2 µm; MG-MCT 155.9 µm), as seen Figure 2 and Table 2. It can be concluded, therefore, that the incorporation of hydroxystearic acid leads to slightly smaller particles, probably due to the impact on the surface tension during particle formation. The determined D_(0.5)_ of the market product is 89.1 µm, which is in line with a size range between 25 and 150 µm reported in the literature [31]. The particle size distribution of the commercial product is considerably broader compared to our formulations (Span Risperdal Consta^®^ 1.165 vs. span values between 0.612 and 0.637), as seen in Table 2.

#### 3.2.2. Scanning Electron Microscopy and Focused Ion Beam Investigations

**Figure 3:**
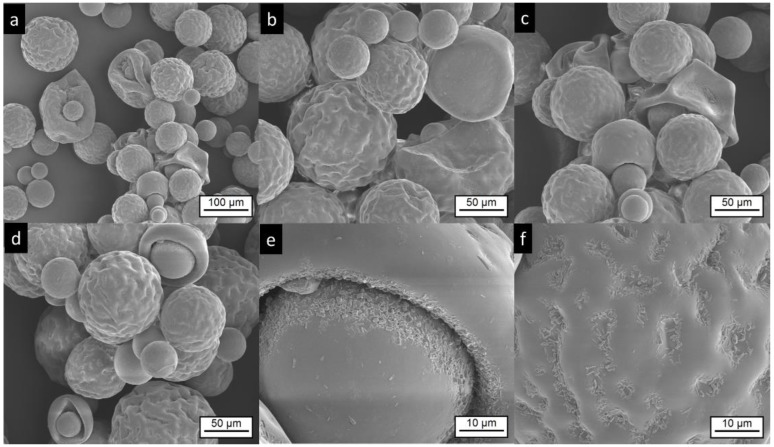
Electron microscopic pictures of Risperdal Consta^®^. The pictures at lower magnifications (**a**–**d**) show the presence of different particle sizes and shapes. Most particles show wrinkled surfaces; some have a smooth surface. High magnification shows the presence of small anisotropic material on the surface (**e**,**f**).

SEM images of Risperdal Consta^®^ are shown in Figure 3. The market product Risperdal Consta^®^ shows a high heterogeneity of particle size, shape, and surface structures. Surprisingly, structures showing partial engulfment, as seen in Figure 3d, and collapsed structures are visible, as seen in Figure 3c. Larger magnification of the surface indicated the presence of anisotropic material, which are most likely drug crystals, as seen in Figure 3e,f.

**Figure 4:**
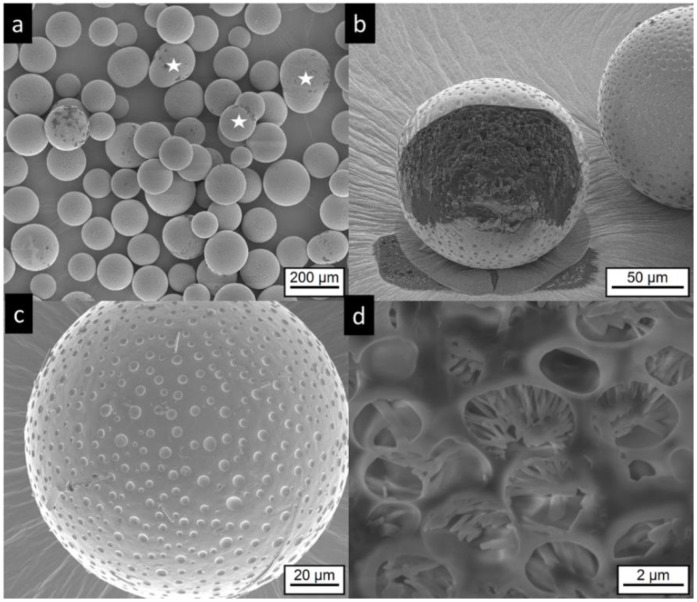
Electron microscopic pictures of MC-MCT microcapsules, (for composition details see Table 1). Intact particles are presented at lower (**a**,**c**) higher magnification. Focused ion beam (FIB) cut particles are shown at lower magnification in (**b**) and after deeper FIB milling and higher magnification in (**d**). The white stars in (**a**) indicate anisotropic particles, which were most likely formed by coalescence of two particles.

The microcapsules and microgels do not show the irregular structures of the commercial product. Most of MCT microcapsules are spherical, some show an elongated shape, which probably results from the coalescence of two particles, as seen in Figure 4a, white stars. The surface of the MC-MCT microcapsules shows a pattern similar to golf balls, as seen in Figure 4b,c. Very rarely, small anisotropic structures are found on the particle surface, as seen in Figure 4c, top middle, which most likely represent risperidone crystals. Cutting of the particles by focused ion beam (FIB) provides insights into the internal structure, as seen in Figure 4b,d. A multicore structure with dimensions in the lower micron range becomes visible. Within the internal structures, anisotropic shapes are visible which most likely present precipitated drug crystals, as seen in Figure 4d.

**Figure 5:**
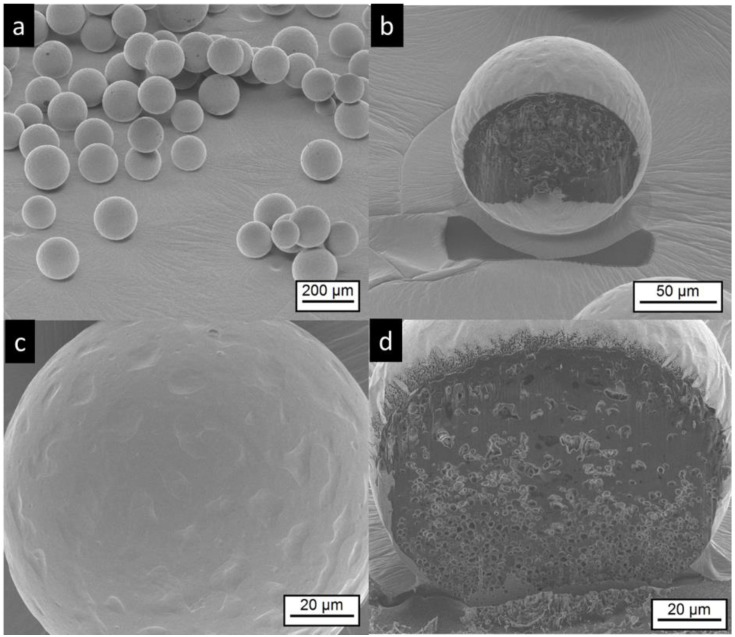
Electron microscopic pictures of MG-MCT microgels (for composition see Table 1). Intact particles are presented at lower (**a**,**c**) higher magnification. FIB cut particles are shown at lower magnification in (**b**) and at higher magnification at (**d**).

The electron microscopic pictures of the corresponding microgel MG-MCT shows spherical particles with a structured surface that appears smoother compared to the MC-MCT surface (Figure 5). In contrast to the MC-MCT microcapsules, we could not detect elongated shapes, as indicated by white stars in Figure 4a, which emerge from the coalescence of two particles. The pictures obtained by FIB cutting of the particles show internal multicore structures with dimensions in the lower micron range, as seen in Figure 5b,d.

**Figure 6:**
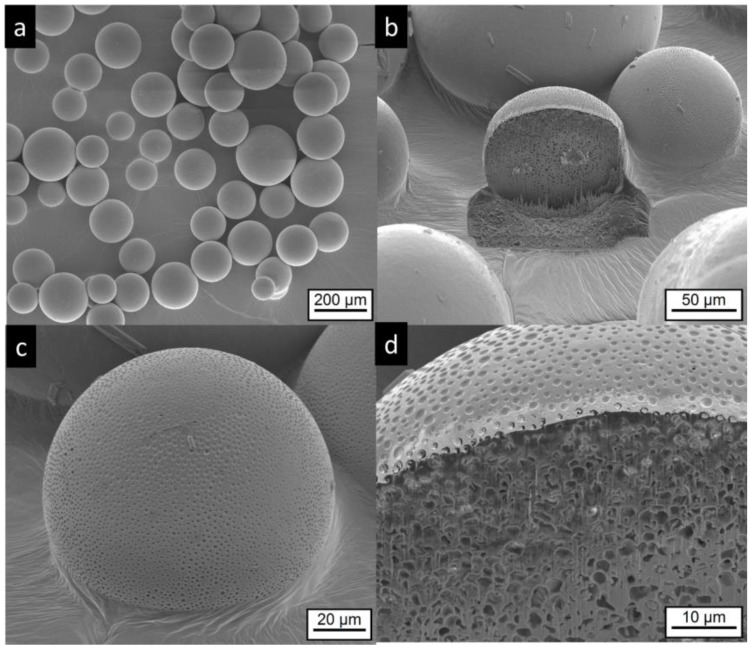
Electron microscopic pictures of MC-IPM microcapsules (composition details are given in Table 1). Intact particles are presented at lower (**a**,**c**) higher magnification. FIB cut particles are shown at lower magnification in (**b**) and at higher magnification at (**d**).

IPM-loaded microcapsules (MC-IPM) have a spherical shape, as seen in Figure 6a. The surface appears similar to the MC-MCT capsules. Very rarely, small crystals can be detected on the particle surface, as seen in Figure 6c. After FIB cutting, multicore structures in the lower micron range become visible, as seen in Figure 6b,d.

**Figure 7:**
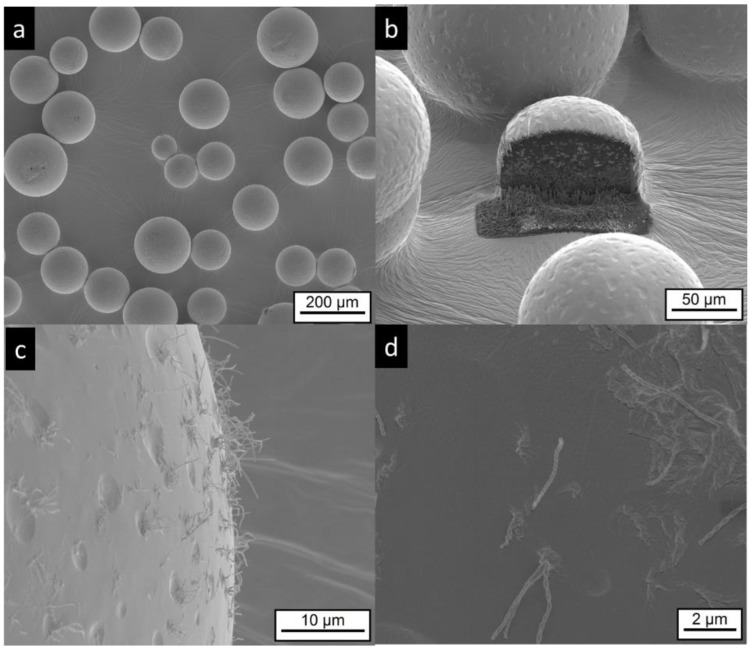
Electron microscopic pictures of MG-IPM microgels (sample composition is given in Table 1). Intact particles are presented at lower (**a**,**b**) and higher (**c**,**d**) magnifications. A FIB cut particle is shown in (**b**). Elongated, “worm like” structures (most likely precipitated hydroxyl-stearic acid) are visible in (**c**,**d**).

The electron microscopy investigation on MG-IPM microgels shows the presence of spherical particles, as seen in Figure 7a. Higher magnifications of the particle surface show surface dips (“golf ball structure”) similar to the MG-MCT microgels. In addition, elongated “hair-like” structures are visible, as seen in Figure 7c,d, which are not detectable in the other samples. Most likely, they are mainly composed of hydroxystearic acid, which is known to induce gel formation by elongated structures. Again, FIB cutting was used to explore the interior of the particles. Multicore microdomains became visible, as seen in Figure 7b, similar to the MG-MCT microgel particles with dimensions in the lower micron range.

**Figure 8:**
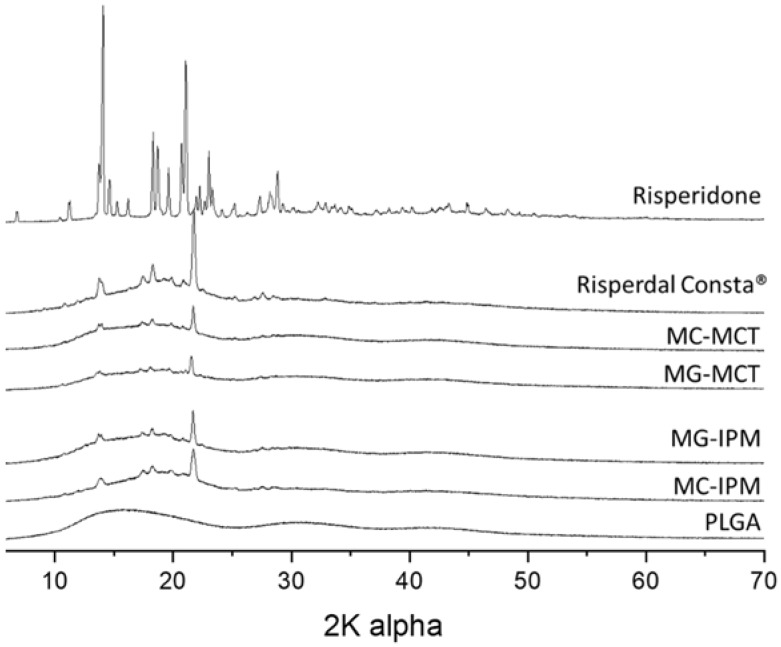
Wide angle X-ray diffractograms of microcapsules, microgels, Risperdal Consta^®^, and the pure drug risperidone.

The crystallinity of the formulations was evaluated by X-ray diffraction. The diffractograms of PLGA, the pure drug risperidone, the commercial product, and the developed microcapsules and microgels are shown in Figure 8. It is known that the drug can exist in different polymorphs [32,33,34]. As expected, the polymer PLGA does not show sharp peaks, but only broad lines, indicating an amorphous state. The pure drug shows multiple sharp peaks. The X-ray diffractograms of the commercial product Risperdal Consta^®^ and the developed formulations show a similar pattern, but they are different from the pure drug. From the measurements, it can be concluded that risperidone exists in all formulations at least partially in the crystalline state. The crystalline state in the formulation is different from the crystal structure of the parent drug, but very similar to the crystal form of risperidone, which has been observed after micronization with supercritical fluids [33].

## 4. X-Ray Nano-Imaging (Nano-CT)

**Figure 9:**
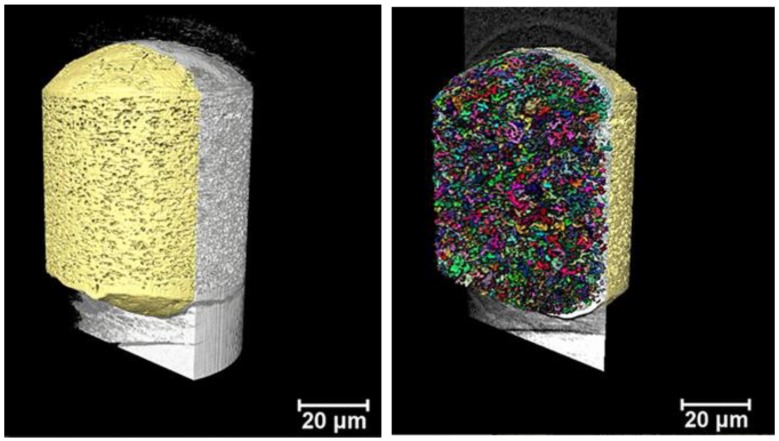
Volumetric representation the inner part of one reconstructed microsphere of Risperdal Consta^®^ imaged with nano-CT. The left picture shows the X-ray signal in gray and the microsphere PLGA matrix pseudocolored in yellow. On the right, the small colored structures represent the dispersed internal spherical structures (ISS), which can be attributed to the dispersed drug particles. The volume of the ISS is about 13% of the total volume of the particle.

**Figure 10:**
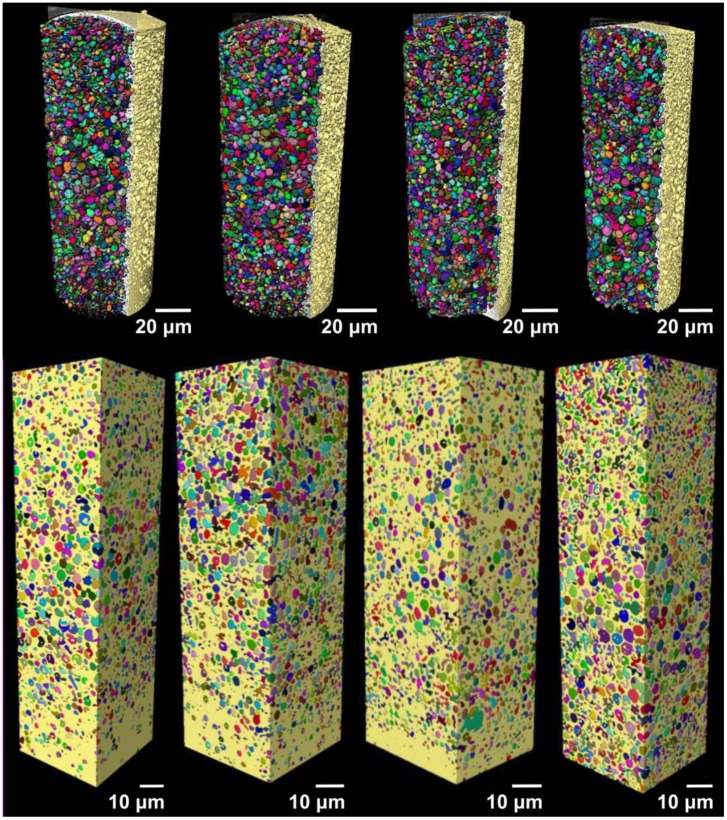
Volumetric representation of the inner part of the microspheres imaged with nano-CT. On the top from left to right, the MC-IPM, MG-IPM, MG-MCT, and MC-MCT microspheres pseudocolored in yellow are shown, with the dispersed internal spherical structures (ISS) represented in different colors. On the bottom, the volume used for the estimations done with nano-CT are shown for all samples.

X-ray imaging comprises a class of nondestructive techniques that are used to visualize the internal structure of different materials. In conventional X-ray imaging, differences in the X-ray absorption cross-section of different materials results in the image contrast [35,36]. Therefore, if the different parts composing one sample show only small differences in the X-ray attenuation, a poor image contrast will be obtained in conventional absorption-based X-ray imaging. This is specially the case for samples composed of elements with low electron density, making the characterization of the structures and visualization of the sample features a very hard task. Alternatively, phase-contrast imaging methods enable the visualization of features of weakly absorbing objects [35,37,38]. X-ray phase-contrast imaging is based on a different physical principle, and the contrast of the image is generated by the detection of the changes in the phase of the wavefront [36,39]. In this work, we imaged the different microspheres using Zernike phase-contrast imaging in a benchtop nano-CT scanner (Xradia Ultra 810). Phase-contrast nano-CT was used to characterize the inner structure of the commercial product Risperdal Consta^®^ (Figure 9) and the developed microcapsules and microgels (Figure 10). Videos Supplementary Videos S1–S5 show the inner 3D structure of the microparticles. The diameter and volume distributions of the internal structures are presented in Supplementary Figures S1 and S2.

All microgels and microcapsules show internal spherical structures (ISS) in the submicrometer range, with sphericity values close to unit. However, the volume percentage of the ISS in the microcapsules is between 35% and 46% and is, therefore, much higher than the volume percentage of the ISS phase in the Risperdal Consta^®^ particles. The ISS phase can be attributed to the dispersed lipid phase for the microgels and microcapsules. In contrast, the ISS consists of risperidone only for the Risperdal Consta^®^ system. A quantitative treatment of the 3D images indicates that the ISS have diameter around 2 µm for lipid-based systems and around 800 nm for the Risperdal Consta^®^ product, as seen in Table 3. The ISS diameter and volume distributions are shown in Figures S1 and S2.

Compared to other imaging techniques, Zernike phase-contrast nano-CT has the advantage of producing high-resolution three-dimensional images of low-absorbing samples, which enables the estimation of the values shown here. As with most high-resolution imaging techniques, nano-CT has the disadvantage of producing images limited to a small field of view, thus, to obtain the images of one single microparticle, it was necessary to image the sample two or three times and stitch the datasets. One of the benefits of phase-contrast nano-CT for imaging low-absorbing samples is that it does not require any special sample preparation procedure or induce any sample damage within the imaged area, in contrast with FIB-SEM. Overall, the results obtained by FIB-SEM correlate well with those obtained by nano-CT, and the same structures were observed. Both methods indicate the presence of a high percentage of internal structures (ISS) with sizes around two micrometers. Therefore, the microcapsules and microgels represent structures III and V of Figure 1, respectively.

## 5. Drug Release

**Figure 11:**
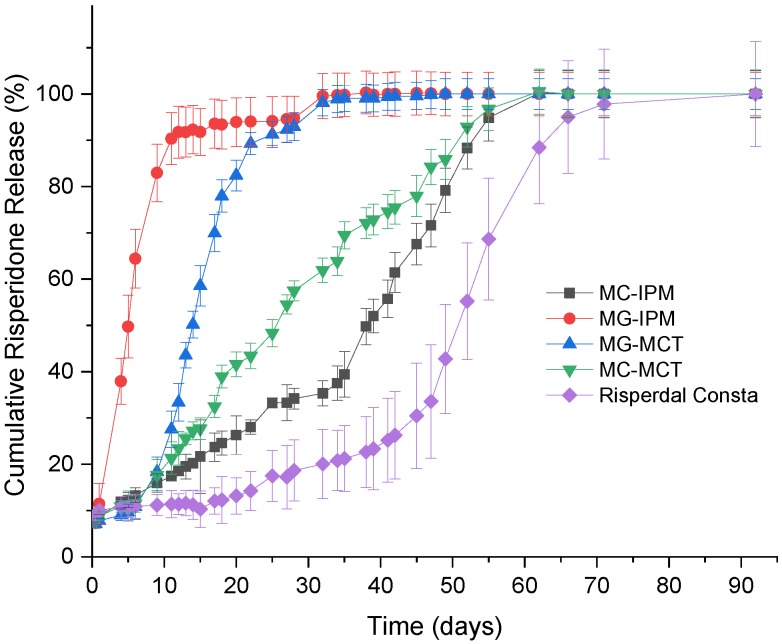
In vitro release profiles of Risperidone microcapsules (MC), microgels (MG), and Risperdal Consta^®^.

The in vitro release of risperidone from the commercial product in the developed formulations is shown in Figure 11. For all formulations, a low percentage of the drug (<10%) is initially released. Most likely, drug molecules on the surface (which were observed by electron microscopy) are released very quickly. After the initial release, there is almost no release detectable for two weeks for Risperdal Consta^®^. Thereafter, the release accelerates. The developed formulations show different release profiles. The fastest release was observed for the MG-IPM microgel. The slowest release rate observed for the lipid formulations was for the MC-IPM microcapsules system. Therefore, it can be concluded that the oleogelator hydroxystearic acid had a strong impact on the release kinetics. The impact of the oleogelator hydroxystearic acid is also visible for the MCT systems, but to a smaller extent. Again, the microparticles with the incorporated oleogel (MG-MCT) release faster compared to the HS-free microcapsules (MC-MCT). The desired release profile was obtained with the MC-MCT microparticles: an almost linear release profile over two months with no lag time has been achieved.

## 6. Conclusions

The results of our study show that lipid–PLGA hybrid systems are interesting alternatives to pure PLGA microparticles. The saturated liquid lipids MCT and IPM were incorporated in the form of microcapsules and microgels. Hydroxystearic acid (HS) was used as an oleogelator. The particle structure was investigated by FIB-SEM and nano-CT imaging. Both methods support the existence of a highly dispersed internal phase with spherical particle sizes of around 2 micrometers. The noninvasive nano-CT imaging enabled the production of 3D representations of the microparticles’ structures in high-resolution images. After image processing and segmentation, it was possible to estimate the amount, size distribution, and sphericity of the internal structures. The presence of hydroxystearic acid accelerated the drug release. The drug release kinetics can be tuned by the lipid composition. An almost ideal release profile for risperidone was achieved with PLGA–MCT microcapsules (MC-MCT). In contrast to Risperdal Consta^®^, no lag time and an almost linear release over 60 days was observed. Therefore, the developed systems are suitable candidates to overcome the need for double medication (injected microparticles and tablets), which is currently needed due to the release lag time of Risperdal Consta^®^.

## Figures and Tables

**Table 1:** Composition and encapsulation efficiency of Risperidone-loaded PLGA microcapsules (MC) and microgels (MG).

Sample	PLGA (mg)	Risperidone [mg]	MCT (mg)	IPM (mg)	HS (mg)	% Encapsul. Efficiency [+/−% SD]
**MC-IPM**	100	50	0	100	0	92.62 [0.94]
**MG-IPM**	100	50	0	90	10	100.82 [5.50]
**MG-MCT**	100	50	90	0	10	96.05 [3.98]
**MC-MCT**	100	50	100	0	0	92.79 [3.23]

MC = microcapsule, MG = microgel, PLGA = poly(lactide-*co*-glycolide), MCT = middle chain triglycerides, IPM = isopropylmyristate, HS = hydroxystearic acid.

**Table 2:** Particle size distributions determined by static light scattering.

Sample	D(0.1) (µm)	D(0.5) (µm)	D(0.9) (µm)	Mean D_(4,3)_ (µm)	Span	Uniformity
**Risperdal Consta^®^**	50.7	89.1	154.5	96.9	1.165	0.36
**MC-IPM**	126.1	172.8	236.3	178.1	0.637	0.202
**MG-IPM**	116.4	157.2	212.6	161.2	0.612	0.193
**MG-MCT**	115.3	155.9	211.8	160.2	0.620	0.196
**MC-MCT**	123.7	169.5	230.8	174.4	0.631	0.197

**Table 3:** Volume, diameter and sphericity of the internal dispersed phase (ISS), assessed by nano-CT.

Sample	Volume ISS	ISS Diameter	ISS Sphericity
Risperdal Consta^®^	13%	0.8 ± 0.2 μm	0.95 ± 0.02
MG-MCT	35%	2.0 ± 0.9 μm	0.8 ± 0.2
MC-MCT	46%	2.2 ± 0.8 μm	0.7 ± 0.1
MC-IPM	39%	1.9 ± 1.3 μm	0.8 ± 0.2
MG-IPM	43%	2.3 ± 1.0 μm	0.8 ± 0.3

## Supplementary Materials

The following are available online at https://www.mdpi.com/1999-4923/11/12/665/s1, Figure S1: Diameter distribution of the internal spherical structures (ISS) determined by nano-CT. Figure S2: Volume distribution of the internal structures (ISS) determined by nano-CT. Video S1: Video of the internal structure obtained by nano-CT for Risperdal Consta. Video S2: Video of the internal structure obtained by nano-CT for MC-IPM. Video S3: Video of the internal structure obtained by nano-CT for MG-IPM. Video S4: Video of the internal structure obtained by nano-CT for MG-MCT. Video S5: Video of the internal structure obtained by nano-CT for MC-MCT.
