# Supplementary material for: Risperidone-Loaded PLGA–Lipid Particles with Improved Release Kinetics: Manufacturing and Detailed Characterization by Electron Microscopy and Nano-CT
Source: Pharmaceutics. 2019 Dec 9;11(12):665. doi: 10.3390/pharmaceutics11120665 (PMC6956012; doi:10.3390/pharmaceutics11120665)
Supplement: Supplementary file 1 [file pharmaceutics-11-00665-s001.zip › Supplement/pharmaceutics-644327-SI - proofed.docx]

Supplementary Materials: Risperidone-Loaded PLGA–Lipid Particles with Improved Release Kinetics: Manufacturing and Detailed Characterization by Electron Microscopy and Nano-CT

Christopher Janich, Andrea Friedmann, Juliana Martins de Souza e Silva,
Cristine Santos de Oliveira, Ligia E. de Souza, Dan Rujescu, Christian Hildebrandt,
Moritz Beck-Broichsitter, Christian E. H. Schmelzer and Karsten Mäder

**Figure 1.** Size distribution of the internal structures estimated by 3D-nano-CT.

**Figure S2.** Volume distribution of the internal structures estimated by 3D-nano-CT.
